# Key microbial taxa play essential roles in maintaining soil muti-nutrient cycling following an extreme drought event in ecological buffer zones along the Yangtze River

**DOI:** 10.3389/fpls.2024.1460462

**Published:** 2024-09-04

**Authors:** Jie Fang, Zihao Liu, Yongcui Deng, Bin Song, Jonathan M. Adams

**Affiliations:** ^1^ School of Geography and Ocean Sciences, Nanjing University, Nanjing, China; ^2^ School of Geography, Nanjing Normal University, Nanjing, China; ^3^ Department of Forest Sciences, University of Helsinki, Helsinki, Finland

**Keywords:** Yangtze drought, 16S rRNA sequencing, microbial community, multifunctionality, nitrogen-cycling

## Abstract

Climatic extremes, especially extreme droughts, are occurring more frequently and profoundly impacting biogeochemical processes. However, the relative importance of microbial communities on soil nutrient cycling and community maintenance under natural extreme drought events remains elusive. During a record-breaking drought in the Yangtze River Basin (YRB) in the summer of 2022, we collected ambient soils and drought-affected bare and vegetated soils in ecological buffer zones from two sites with similar soil and vegetation characteristics along the YRB, and examined the relative contribution of soil bacterial communities in supporting multi-nutrient cycling index (MNCI) involving carbon-, nitrate- and phosphorus-cycling and their associations with microbial network. Extreme drought decreased (*p* < 0.05) bacterial α-diversity but increased MNCI in vegetated soils at both sites, while both remained unchanged (*p* > 0.05) in bare soils, possibly as a result of vegetation releasing rhizodeposits under drought which selectively recruited bacterial communities. Bacterial community compositions were shifted (*p* < 0.05) only in vegetated soils, and they exerted more influence than α-diversity on soil MNCI. Notably, the Anaerolineae, identified as a biomarker enriched in vegetated soils, had close associations with enzyme activities and soil MNCI at both sites, suggesting their potential recruitment by vegetation to withstand drought. Furthermore, key ecological clusters (Module 1) in bacterial co-occurrence networks at both sites supported (*p* < 0.05) higher MNCI, despite no substantial variation in network structure due to drought. Specifically, the most important taxa within Module 1 for predicting soil MNCI revealed by random forest modeling analysis (R^2^ = 0.44 - 0.63, *p* < 0.001), such as B1-7BS, SBR1031 and Nocardioides, could be deeply involved in soil nitrogen-cycling, suggesting an essential role of specialized interactions of bacterial communities in maintaining soil multifunctionality. Overall, this study demonstrates that changes in biomarkers and functional taxa under extreme drought may better reflect the biological mechanisms involved in microbial communities impacting ecosystem function, which may aid in forecasting the ecological consequences of ongoing climate change in the ecological buffer zones along the YRB.

## 1 Introduction

Over recent decades, the steadily rising temperature due to global warming has led to an upsurge in climate extremes, occurring with greater frequency and intensity (Zhang et al., 2022a; Xu et al., 2023), which have caused varying degrees of devastating impacts on the natural ecological balance and human health (Marvel et al., 2019). Among these natural hazards, droughts are projected to become more frequent, longer, and more severe in many regions worldwide. They can develop quickly into severe droughts (e.g., flash droughts) within weeks (Christian et al., 2023; Yuan et al., 2023), increasingly threatening terrestrial ecosystem functioning and stability. In particular, biodiversity loss due to droughts arising from anthropogenic environmental changes, such as global warming and reduced precipitation, disturbs the functioning of natural ecosystems and diminishes their capacity to provide services, both quantitatively and qualitatively (Liu et al., 2023; Zhang et al., 2024a). Therefore, an improved knowledge of how belowground biodiversity responds to drought and the underlying mechanisms are necessary to predict shifts in ecosystem services or functions in the face of increasingly frequent droughts (Preece et al., 2019; Buscardo et al., 2021; Weides et al., 2024).

Soil microbiota are essential component that underpin ecosystem functioning and soil biogeochemical processes. They play crucial roles in various ecosystem processes such as primary production (e.g., through plant-microbe interactions), nutrient cycling and decomposition, as well as mediate the ecosystem resistance to anthropogenic pressures and climate changes (Steudel et al., 2012; Hu et al., 2021; Jiao et al., 2022a). However, soil microbial community structure and function are highly sensitive to environmental changes (Jiao et al., 2021; Fu et al., 2022), and any alterations can impact biodiversity-ecosystem function relationships and subsequent ecosystem services, such as nutrient cycling and plant community dynamics. Given the importance of soil microbial diversity for ecosystem multiple functions, advancing our understanding of how soil microbiome supports ecosystem functioning under global change scenarios, particularly increasingly frequent droughts, is imperative for developing effective management strategies to maintain ecosystem services (Jiao et al., 2019; 2022b; Zhang et al., 2024a).

Based on varying spatial and temporal scales, drought can be defined as a persistent water deficit and can be categorized into meteorological drought, agricultural drought, hydrological droughts and socio-economic drought (Xu et al., 2023; Yuan et al., 2023). Extensive studies have investigated the effect of drought on the soil microbiota and related ecosystem functions by manipulating precipitation reduction and warming (i.e., increased temperature) at local scales or by sampling along natural aridity gradients at large scales. For instance, Fu et al. (2022) demonstrated that soil microbial communities exhibited greater sensitivity to intense drought (i.e., 100% reduction in precipitation for 2 months) than chronic drought (66% reduction in precipitation for 4 months). Wu et al. (2022) found that experimental warming (+3°C above ambient temperature) decreased microbial richness (including bacteria, fungi and protists), suggesting heightened vulnerability of associated ecosystem functions and services in a warmer climate. Additionally, Zhang et al. (2024b) concluded that simplified microbial network (i.e., decreased network complexity) reduced microbial community stability and soil functionality with increasing aridity gradients. However, it is important to note that these studies above primarily focused on grassland ecosystems under manipulated conditions. Despite the improved knowledge of the ecological consequences of droughts from such studies, there is a lack of systematic assessment of the effects of natural extreme drought events on the microbial structure and ecosystem functioning in complex natural ecosystems (Mishra et al., 2021; Liao et al., 2023), especially in light of increasingly frequent drought occurring globally.

In the summer of 2022, the most severe drought in recent history hit the Yangtze River Basin (YRB), leading to the drying up of numerous tributaries and great crop losses along YRB, as documented by the Ministry of Water Resources of the People’s Republic of China (http://www.mwr.gov.cn), news media (https://www.bbc.co.uk/news/62751110) and scientific publications (Wang et al., 2023a; Xu et al., 2023). Against this backdrop, we conducted a study by sampling the drought-affected exposed bare soils and adjacent vegetated soils from ecological buffer zones at two sites with similar soil conditions and vegetations along YRB, with wet soil in waterward zones as the ambient soil. Given the strong responses of plants to drought, such as increased root exudates and root extension (Jones et al., 2004; Weides et al., 2024), it was hypothesized that compared to exposed bare soil, drought would exert a greater impact on microbial communities in vegetated soils, and equally on the soil functioning associated with them. Therefore, the objects of this study were: i) to explore changes in microbial community structure, including diversity, community composition and co-occurrence patterns, in response to drought; and ii) to evaluate the ecological roles of microbial communities on maintaining soil functionality, particularly in multi-nutrient cycling, during the drought event. Microbial community analysis employed 16S rRNA gene sequencing, while soil functionality was characterized by measuring multiple soil functions related to soil carbon (C)-, nitrogen (N)- and phosphorus (P)-cycling. This work may contribute to predict changes in biodiversity-driven ecosystem functioning and inform effective management strategies to optimize ecosystem service provisioning in the ecological buffer zones along YRB under global change scenarios.

## 2 Materials and methods

### 2.1 Study sites and samples collection

From July to September 2022, the entire YRB experienced an unprecedented extreme drought and heatwave, with the highest average temperatures and number of hot days (maximum temperatures > 35°C) over this period since 1961 when meteorological observations were available (Ni et al., 2024). During this period, precipitation declined sharply by ~ 46% and ~ 36% compared with that in 2020 and 2021 (Xu et al., 2023), respectively, which profoundly influenced the proper and stable functioning of ecosystems (both natural vegetation and human-managed crops) along YRB (Wang et al., 2023a; Xu et al., 2023). In Nanjing section of YRB, two sampling sites were distinguished and selected due to the unique ecological buffer zones developed by the extreme drought leading to the substantial drop in water-level (**Supplementary Figure S1**). Specifically, one sampling site (118°41′E, 32°04′N) was located in Pukou district (PK), featuring drought-affected bare soils (i.e., originally derived from deposited river sediments), young reeds (*Phragmites australis* (Cav.) Trin. ex Steud), grasses (mainly *Carex tristachya* Thunb.) and old reeds, respectively, on the natural slopes from the river to the shoreline. Similarly, another site (118°55′E, 32°11′N) was in Luhe district (LH), but with bare soils, young reeds, old reeds and mixed trees (mainly *Populus* L. and *Salix babylonica* L.), respectively, from the river to the shoreline. The plants are typical vegetation in wetland along the YRB and their distribution are strongly affected by the intensification of drought and the extension of the dry season (Zhang et al., 2024c).

Soil sampling was conducted in early September 2022. Within each zone, due to the limitations of finite successional zones, soil samples were collected from three independent quadrats measuring 1 × 1 m, space 20 m apart horizontally from each other. To minimize edge effects, all quadrats were located along the middle elevation line of each zone. Within each quadrat, the topsoil (0 - 2 cm) was collected using a 5 cm soil core, taken from the four corners and the center, and then thoroughly mixed to represent one sample of this zone. The topsoil (0 - 2 cm) from the waterward zone, which remained virtually unaffected by drought and was collected as the ambient soil (i.e., the control). This took into account the fact that topsoil across the limited successional zones tended to be homogenized by the reciprocating tidal flows during the regular period (Wang et al., 2023b). A total of 30 soil samples from two sampling sites were collected and transported to the laboratory in an ice box and sieved through a 2.0-mm mesh. The majority of each soil sample was stored at 4°C for the analysis of soil factors, while a portion was stored at −40°C for subsequent DNA extraction.

### 2.2 Quantifying the soil multi-nutrient cycling index

The present study measured 14 soil variables, 11 of which were used to estimate the soil multi-nutrient cycling index (MNCI). Specifically, basic soil properties included soil water content (SWC), pH, conductivity (Cond), salinity (Sal), total carbon (TC), total nitrogen (TN), total phosphorus (TP), available nitrogen (AN) and available phosphorus (AP) were measured using the methods described previously (Fang et al., 2022). Extracellular enzyme activities including β-glucosidase (BG) and β-Xylosidase (BX) involved in C-cycling, leucine aminopeptidase (LAP) and N-acetyl-β-glucosaminidase (NAG) involved in N-cycling, and alkaline phosphatase (ALP) involved in P-cycling processes were measured using a microplate fluorometric assay (Dong et al., 2022).

Among these properties, soil pH, Cond and Sal were not included when calculating the MNCI (Qiu et al., 2021), as other variables can provide direct support for nutrient cycling and the establishment of nutrient pools. To obtain a quantitative soil MNCI value for each sample, the variables were normalized (square root transformed) and standardized using the Z-score transformation. Hereafter, these standardized ecosystem functions were then averaged as the MNCI (Delgado-Baquerizo et al., 2016; Jiao et al., 2019; Sun et al., 2024).

### 2.3 DNA extraction and amplicon sequencing

Soil DNA was extracted from 0.5 g of fresh soil with FastDNA Spin Kits for Soil (MP Biomedical, Santa Ana, CA) based on the manufacturer’s instructions. The hypervariable V4-V5 regions of bacterial 16S rRNA genes were amplified with 515F (5C-GTGCCAGCMGCCGCGGTAA-3’) and 919R (5’-CCGTCAATTCMTTTRAGTTT-3’) primers. Detailed information about PCR mixtures and conditions for 16S rRNA gene amplification were described previously (Song et al., 2023). Purified amplicons were pair-end sequenced (2 × 300 bp) on an Illumina MiSeq platform at Majorbio (Shanghai, China). Paired-ends raw sequences were merged and filtered using USEARCH v10.0 (Edgar, 2010), following the USEARCH pipeline (http://www.drive5.com/usearch/manual/uparse_pipeline.html). Operational taxonomic units (OTUs) were clustered at 97% identity using UPARSE algorithm. Representative sequences were classified against SILVA v138 database to obtain taxonomic information for each OTU. A total of 958,840 sequences were obtained from 30 samples, and the number of considered sequences per sample were normalized based on minimum sequence size for downstream analyses. The resulting sequencing data are publicly available at NCBI SRA with accession numbers PRJNA1139050.

### 2.4 Statistical analysis

Statistical analyses were performed in SPSS 22.0. Prior to analysis, data was tested for the normality and homogeneity, and log-transformed if necessary. Significant differences in abiotic and biotic variables among zones were determined using Duncan’s multiple range test (*p* < 0.05) following one-way ANOVA. Principal component analysis (PCA) and principal coordinates analysis (PCoA) were performed to visualize variation in multiple ecosystem functions and bacterial community composition across successional zones by drought, respectively. Permutational multivariate analysis of variance (PERMANOVA) was used to test significant differences in these variables among zones. Functional profiling of bacterial communities among zones was predicted using the Tax4Fun2 package (Wemheuer et al., 2020). Linear discriminant analysis (LDA > 2) effect size (LEfSe) was used to elucidate significant differences in the abundances of bacterial taxa (i.e., biomarker) and predicted functions among zones, using the microeco package (Liu et al., 2020). Redundancy analysis (RDA) was used to determine the importance of environmental variables in shaping bacterial communities. Prior to RDA, the OTU data was Hellinger-transformed, and collinear explanatory variables were removed until all variables with ‘vif’ < 10 (Liu et al., 2024). The quantitative contribution of selected variables was then examined using the rdacca.hp package (Lai et al., 2022). Structural equation model (SEM) was used to achieve a deeper understanding of the extreme-drought induced direct and indirect effects on soil MNCI, using AMOS v24.0. Causal relationships between predictive variables within SEM were based on prior knowledge, and all selected variables were treated as independently observed variables. Model probability was evaluated by following criteria (Fan et al., 2016): low χ^2^ values (P > 0.05), high the comparative fit index (CFI) ≥ 0.95, and low root mean square error of approximation (RMSEA) < 0.06.

Co-occurrence networks of bacterial communities were constructed using the SPIEC-EASI, a robust method against community compositionality bias (Kurtz et al., 2015). OTUs occurring in ≥ 6 samples with relative abundances ≥ 0.05% were selected for network analyses. These filtering criteria can avoid the biased effects of rare taxa within a site in network analysis. Networks of robust correlations were defined as |r| > 0.60 and *p* < 0.05, and were visualized using the Fruchterman-Reingold layout in Gephi v0.9.2. Network topological properties including node number, edge number and average degree were calculated using the igraph package (http://igraph.org).

## 3 Results

### 3.1 Changes in soil properties, enzyme activities, and multi-nutrient cycling across drought-affected zones

**Figure 1:**
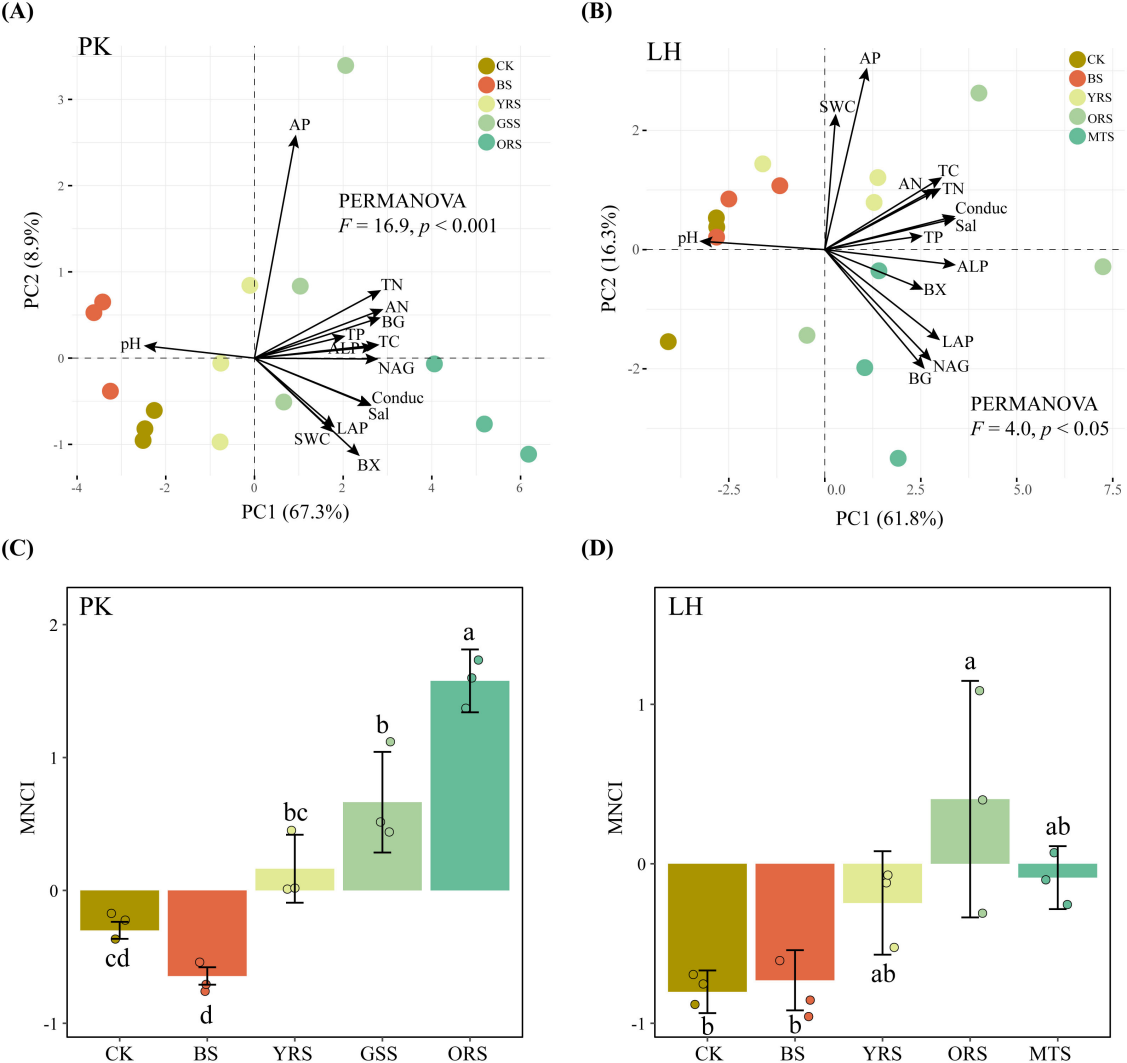
Distribution of basic soil properties and enzyme activities across drought-affected zones by principal component analysis **(A, B)** and comparison of soil multi-nutrient cycling index **(C, D)** at PK and LH sites, respectively. Different letters in the bars in panel **(C, D)** indicate significant differences (*p* < 0.05), and error bars indicate the standard deviations (n = 3). CK, wet soil in waterward zone; BS, bare soil; YRS, soil with young reeds; GSS, soil with grasses; ORS, soil with old reeds; MTS, soil with mixed trees; SWC, soil water content; Conduc, conductivity; Sal, salinity; TC, total carbon; TN, total nitrogen; TP, total phosphorus; AN, available nitrogen; AP, available phosphorus; BG, β-glucosidase; BX, β-Xylosidase; LAP, leucine aminopeptidase; NAG, N-acetyl-β-glucosaminidase; ALP, alkaline phosphatase.

Soil properties varied significantly across zones at both sites (**Supplementary Tables S1, S2**), as demonstrated by the PERMANOVA (**Figures 1A, B**). Compared with the wet soil in the waterward zone (i.e., CK), drought-affected vegetated soils (YRS, ORS, GSS and MTS) exhibited higher TC, TN, TP and AN contents, conductivity (Cond) and salinity (Sal) concentration, and lower pH at both sites, while lacking differences between CK and drought-affected bare soil (BS). At both sites, the activities of BG, BX, LAP, NAG and ALP increased steadily and significantly on natural slopes from CK to vegetated soils (except for BS). Similarly, the MNCI increased steadily and significantly along the natural slopes, while showing a slight decrease in BS at PK and lacking difference between CK and BS at LH (**Figures 1C, D**).

### 3.2 Bacterial community structure and its contribution to multi-nutrient cycling

**Figure 2:**
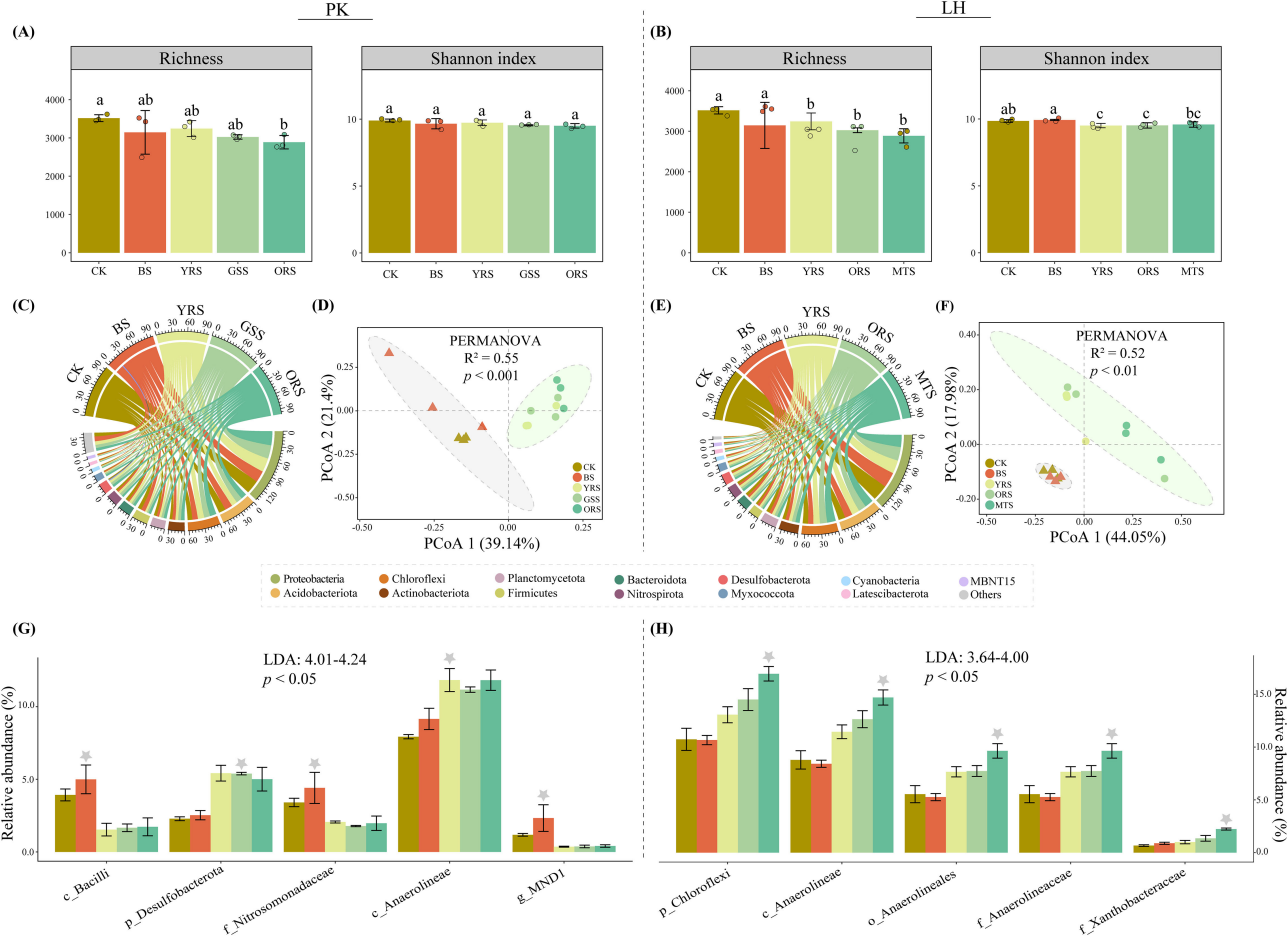
Analysis of Richness and Shannon indices of the bacterial community **(A, B)**, relative abundances at the phylum level **(C, E)** and community composition by principal coordinate analysis **(D, F)** across drought-affected zones at PK and LH sites, respectively. In panel **(A, B)** different letters in the bars in panel **(C, D)** indicate significant differences (*p* < 0.05), and error bars indicate the standard deviations (n = 3). In panel **(C, E)** others indicate these relative abundances < 1%. Linear discriminant analysis (LDA) effect size analysis of relative abundances of bacterial taxa at varying taxonomic levels among zones at PK and LH sites **(G, H)**. Only the top five taxa with the largest differentiation size were shown (LDA > 2). Grey asterisks topped in the bar indicate zones of significant enrichment with the greatest LDA score. p, phylum; c, class; o, order; f, family; g, genus.

In contrast to MNCI, bacterial richness decreased steadily and significantly from CK to vegetated soils at both sites (**Figures 2A, B**). However, there was no significant difference in Shannon among zones at PK, whereas it decreased significantly in vegetated soils at LH compared to CK. The predominant phylum at both sites was Proteobacteria (PK: 24.6%-29.9%; LH: 24.2%-31.8%), followed by the Acidobacteriota (PK: 11.9%-16.3%; LH: 13.6%-17.0%) and Chloroflexi (PK: 10.2%-13.6%; LH: 10.7%-16.9%) (**Figures 2C, E**). Principal coordinate analysis (PCoA) showed that bacterial community composition differentiated significantly among zones at both sites (PK: R^2^ = 0.55, *p* < 0.001; LH: R^2^ = 0.52, *p* < 0.01) (**Figures 2D, F**), especially between CK and vegetated soils, but lacked differences between CK and BS and between vegetated soils (**Supplementary Table S3**).

**Figure 3:**
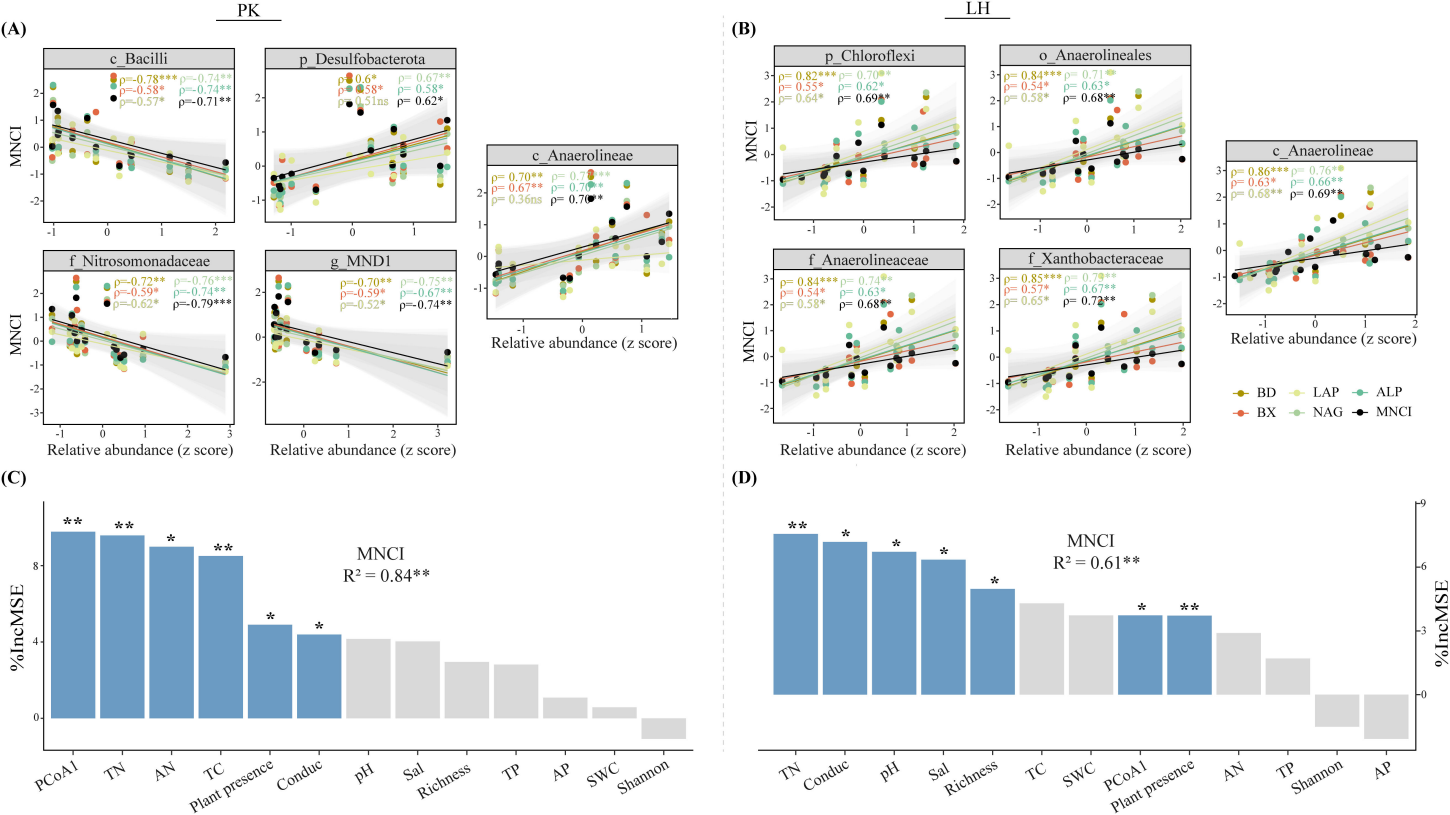
Relationships between the relative abundances of biomarkers and multi-nutrient cycling index and enzyme activities **(A, B)** at PK and LH sites. ρ indicates Spearman’s correlation coefficients (ns, *p* > 0.05; *p < 0.05; **p < 0.01; ***p < 0.001). The shaded area shows the 95% confidence interval of the fit. Random forest analysis showing the major drivers of MNCI **(C, D)** at PK and LH sites. MSE is the mean square error. *p < 0.05, **p < 0.01 on the bar indicate a significant effect of a factor on MNCI.

LEfSe analysis (LDA > 2) revealed the top five bacterial taxa (i.e., biomarker) ranking from high (phylum) to low (genus) taxonomic level that significantly differed among zones (**Figures 2G, H**). Specifically, the Bacilli class (LDA = 4.24), Nitrosomonadaceae family (LDA = 4.12) and MND1 genus (LDA = 4.01) were significantly enriched in BS while the Desulfobacterota phylum (LDA = 4.19) and Anaerolineae class (LDA = 4.12) were significantly enriched in vegetated soils at PK. At LH, all top five taxa were enriched in MTS, and notably, with Anaerolineae present at both sites. The relative abundances of these biomarkers were significantly correlated with MNCI and most enzyme activities at both sites (**Figures 3A, C**), implying their indicative roles in driving changes in MNCI. Furthermore, random forest (RF) analysis revealed bacterial community composition (PCoA1), soil TN and plant as significant predictors for MNCI at both sites (**Figures 3B, D**), with PCoA1 and TN being the strongest predictors for MNCI at PK and LH, respectively.

### 3.3 Effects of biotic and abiotic factors on bacterial communities and multi-nutrient cycling

**Figure 4:**
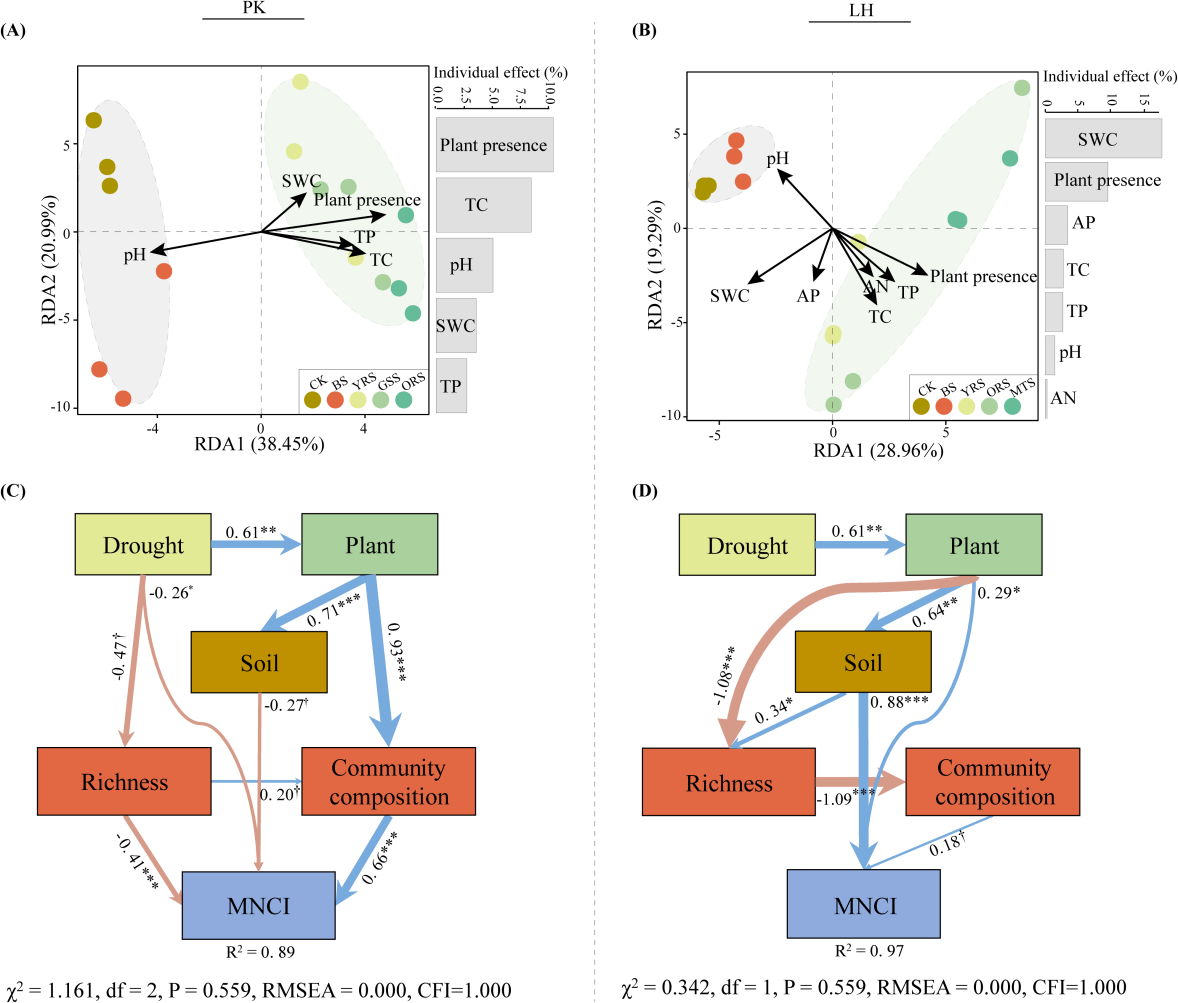
Redundant analysis (RDA) of environmental factors structuring bacterial community composition **(A, B)** at PK and LH sites. Colinear environmental variables were removed until all variables showed a ‘vif’ < 10. Bars on the right panel of RDA plots are the individual effects of each selected variable. The variable of plant presence is converted to a binomial (presence = 1; absence = 0). Using fitted structural equation models (SEM) to elaborate the direct and indirect effects of the extreme-drought on multi-nutrient cycling **(C, D)** at PK and LH sites. Arrow width indicates the strength of the standardized effect, and values in each arrow indicate the path coefficients (^†^, *p* < 0.10; *p < 0.05; **p < 0.01; ***p < 0.001), with nonsignificant effects (*p* > 0.10) removed from the model. Blue and pink lines indicate the positive and negative effects, respectively, and R^2^ values indicate the proportion of variance explained. Bacterial community composition is represented by the PCoA axis1, and soil variable is represented by the PCA axis1.

Redundant analysis (RDA) revealed that soil (SWC, pH, TC and TP) and plant presence strongly influenced bacterial communities at PK, with plant presence being the dominant factor (**Figure 4A**). Conversely, at LH, these factors, along with soil AP and AN, strongly influenced bacterial communities (**Figure 4B**), with SWC emerging as the dominant factor. Structural equation models (SEM) were performed to examine the direct and indirect effects of extreme drought on MNCI. In total, the SEM explained 89% and 97% of variations in MNCI at PK and LH (**Figures 4C, D**), respectively. In SEM of PK, the drought had a direct effect on MNCI (λ = −0.26, *p* < 0.05), and also indirectly affected MNCI through changes in bacterial richness (λ = −0.41, *p* < 0.001), and changes in soil properties (λ = −0.27, *p* < 0.10) and bacterial community composition (λ = 0.66, *p* < 0.001) mediated by plant presence. In contrast, in the SEM of LH, the drought had no direct effect on MNCI, but indirectly affected MNCI through the change in plant (λ = 0.29, *p* < 0.05), and change in soil (λ = 0.88, *p* < 0.001) mediated by plant presence.

### 3.4 Co-occurrence patterns and functional profiles of bacterial communities

**Figure 5:**
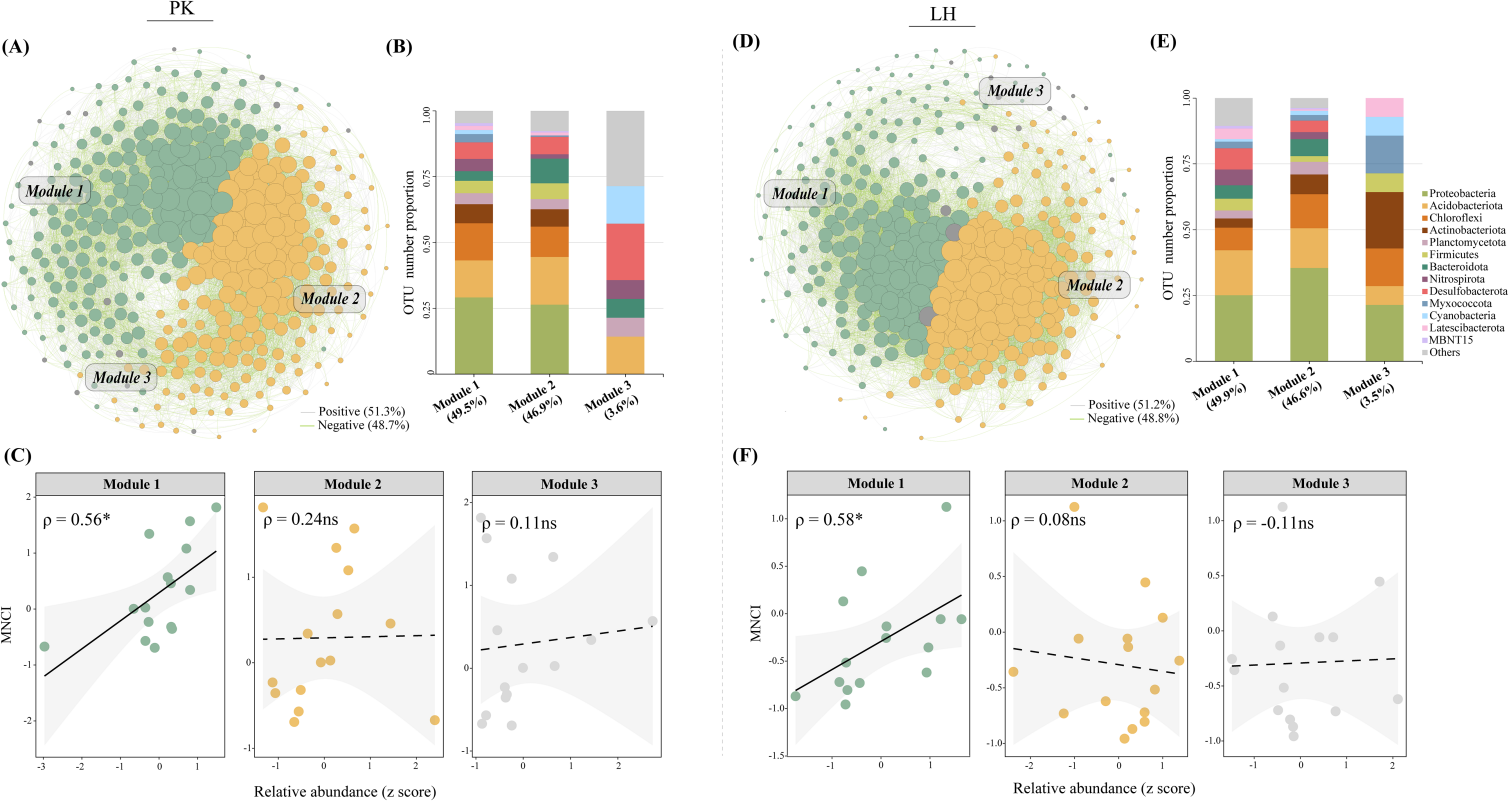
Network diagrams with nodes colored according to the main ecological clusters (Modules 1–3) **(A, D)** and OTU number proportion of the bacterial communities in the main ecological clusters **(B, E)** at PK and LH sites, respectively. The size of each node is proportional to the number of degrees. Relationships between the relative abundances of main ecological clusters and multi-nutrient cycling index **(C, F)** at PK and LH sites. ρ indicates Spearman’s correlation coefficients (ns, *p* > 0.05; *, *p* < 0.05). The shaded area shows the 95% confidence interval of the fit.

Co-occurrence network analyses were conducted to reveal the potential bacterial association patterns along drought-affected zones. The topological features including the nodes, edges and average degree were slightly decreased in BS and ORS at PK, and similar decreases were identified in ORS and MTS at LH compared to CK (**Supplementary Table S4**). There 11 and 6 key-stone nodes were identified in networks of PK and LH, respectively (**Supplementary Figure S2**). Notably, three prevalent ecological clusters (Modules 1-3) were identified in the respective networks at both sites (**Figures 5A, D**). These clusters were mainly composed of Proteobacteria (PK: 26.4%-29.2%; LH: 25.1%-35.5%), Acidobacteriota (PK:14.1%-18.1%; LH:15.1%-17.1%) and Chloroflexi (PK:11.5%-14.1%; LH:8.5%-12.9%) (**Figures 5B, E**). Module 1 accounted for 30.8% and 32.0% of total relative abundance at PK and LH sites, respectively, and both positively correlated with MNCI (**Figures 5C, F**).

**Figure 6:**
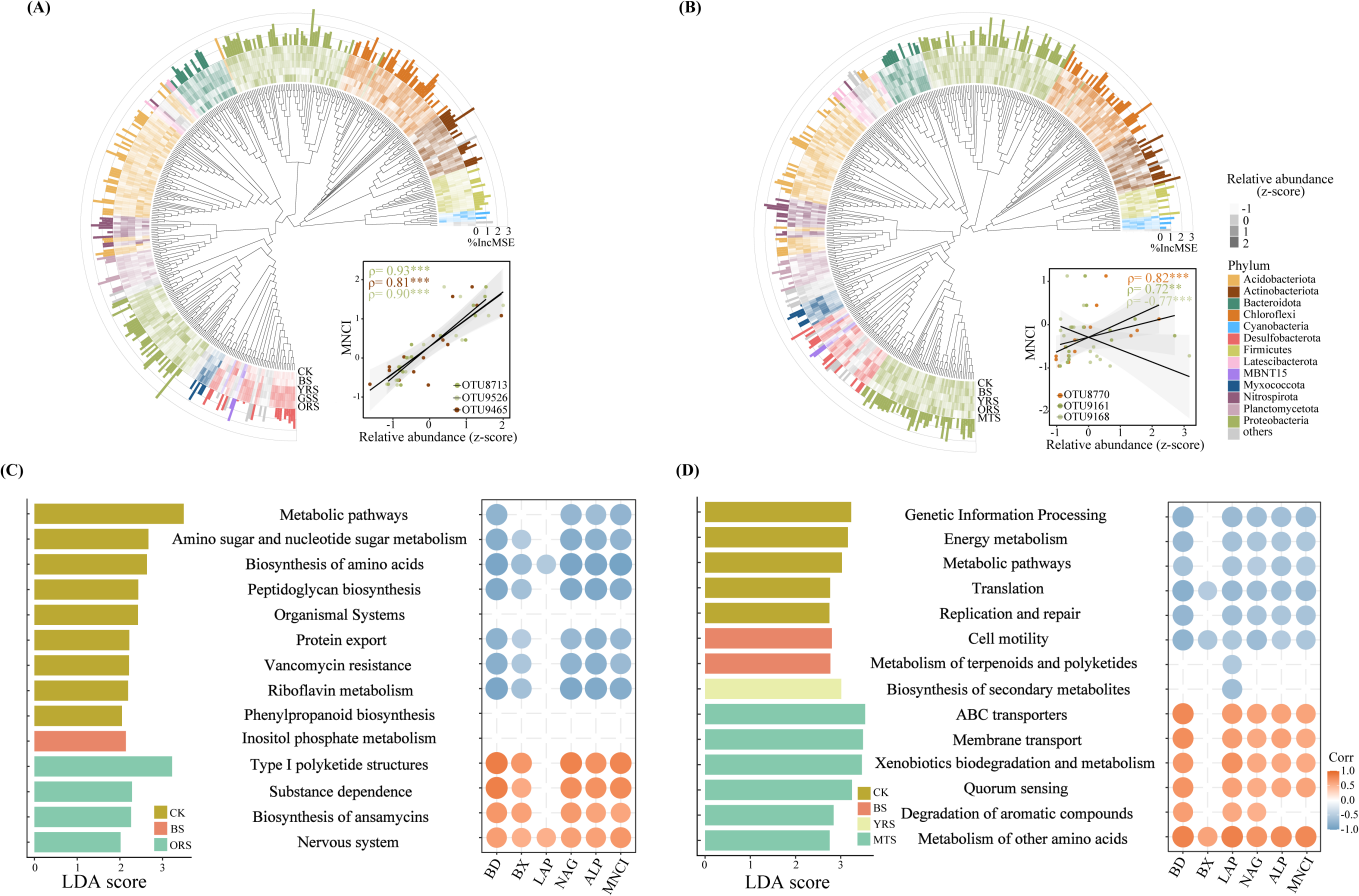
Phylogenetic distributions for bacterial communities of Module 1 across drought-affected zones and their relative importance for multi-nutrient cycling index (MNCI) at PK and LH **(A, B)** sites. Colors are corresponding to individual phyla. The colored heatmap shows the changes in the relative abundance of bacterial OTUs within Module 1 of networks, and the length of colored blocks represents its relative importance for predicting MNCI by random forest analysis, with the most important bacterial OTUs (top three) showing their relationships with MNCI. MSE indicates the mean square error, with MSE ≤ 0 not shown. Linear discriminant analysis (LDA) effect size analysis of relative abundances of predicted functions (by Tax4Fun2) of bacterial communities within Module 1 (left) and their significant correlations (*p* < 0.05) with MNCI and enzyme activities (right) at PK and LH sites **(C, D)**. The top functions with the largest differentiation size were shown (LDA > 2) at LH.

There were strong variations in the relative abundances of bacterial species (at OTU-level) in Module 1 among zones of both sites (**Figures 6A, B**), with more enriched in vegetated soils (**Supplementary Figure S3**). RF analysis revealed that a significant portion of the variation in MNCI could be explained by these OTUs within Module 1 (PK: R^2 ^= 0.63, *p* < 0.001; LH: R^2 ^= 0.47, *p* < 0.001). Among these, OTU8713 (norank_f_B1-7BS), OTU9526 (norank_o_SZB30) and OTU9465 (g_Nocardioides) were the strongest predictors for MNCI and were also significantly correlated with MNCI at PK, while these were OTU8770 (norank_o_SBR1031), OTU9161 (g_Dongia) and OTU9168 (norank_f_A0839) at LH. Functional prediction of Module 1 by the Tax4Fun2 showed that more functions were enriched in CK (level 1-3) compared to these drought-affected zones at both sites by LEfSe analysis (LDA >2) (**Figures 6C, D**; **Supplementary Figure S4**). The metabolic pathways (level 3) were enriched in CK at both sites and negatively correlated with enzyme activities and MNCI, while the functions enriched in ORS (at PK) or MTS (at LH) positively correlated with enzyme activities and MNCI.

## 4 Discussion

### 4.1 Drought-affected vegetated soils enhance multi-nutrient cycling in the ecological buffer zones along YRB

Extreme-drought events are becoming more frequent globally as climate change intensifies and are transitioning to flash droughts (rapid-onset droughts) characterized by abnormally high evapotranspiration and quick depletion of soil water (Yuan et al., 2023), which profoundly threatens the terrestrial ecosystem functions (Berdugo et al., 2020). For instance, the significant decline in soil water content (SWC) due to drought exerted pose selective pressure on plant growth and belowground microbial species, detrimentally affecting biogeochemical processes (Buscardo et al., 2021; Zhang et al., 2024b). Here, the examination of two sites with distinct soil conditions and vegetative covers exhibited a consistent drought-induced enhancement in soil multi-nutrient cycling in vegetated soils (**Figures 1C, D**). This finding contrasted with Sun et al. (2024), who suggested that the multi-nutrient cycles were decreased in the arid zone compared with the dry-wet cycling zone in lakeshore wetland. One explanation for the difference was that drought only induced a slight reduction of SWC in the soils due to the presence of vegetation (**Supplementary Tables S1**, **S2**). Vegetation can reduce water evaporation through shading effects (Raz-Yaseef et al., 2010) and form symbiotic relationships with arbuscular mycorrhizal fungi, which promote water-stable aggregate formation and moisture retention (Rillig, 2004; Liu et al., 2021). The positive correlations between SWC and most nutrients and enzyme activities further support the notion that vegetation can help maintain soil functionality under drought stress (**Figures 1A, B**).

Alternatively, plants could actively or passively release rhizodeposits (carbon compounds from living plant roots) into the surrounding soil under drought stress, which certainly increases belowground nutrient cycling and turnover (e.g., proliferation of microorganisms) (Jones et al., 2004). Although the loss of carbon from the root system may initially seem like a poor strategy for plants, rhizodeposition may be beneficial to plants since it increases plant uptake of nutrients from the rhizosphere, mainly through the stimulation of microorganisms, thus aiding in coping with drought stress (Jones et al., 2004; Preece and Peñuelas, 2016). Indeed, plants may have little control over carbon loss during drought stress, even if these lost compounds sometimes seem to play a minor role in mitigating stress (Jones et al., 2004). However, the response of drought on rhizodeposition per individual was highly variable - leading to changes from neutrality to increases or decreases - and exhibited no relationship with drought intensity, as summarized by Preece and Peñuelas, (2016). Of particular note, the concomitant reduction in plant biomass under very severe drought may lessen the amount of rhizodeposits into the soil (Brunner et al., 2015; Preece and Peñuelas, 2016). Therefore, advancing researches on rhizodeposition are needed to assess which habitats (e.g., vegetation along YRB) are most at risk from increasingly frequent drought.

Interestingly, despite a sharp decrease in SWC compared to vegetated soils, the drought did not significantly alter the multi-nutrient cycling in bare soils at both sites. Topography plays a significant role in influencing soil biogeochemical cycling via its effects on aboveground plant diversity and belowground microbial communities (Qiu et al., 2024; Wang et al., 2024). The deposition process (e.g., transport of nutrients) along the slopes may have obscured the drought-induced effects, especially given the long-term exposure of soils in ecological buffer zones to erosive effects from reciprocal tidal flows. Nonetheless, the soil multi-nutrient cycling in bare soils at PK was still decreased by drought (**Figure 1C**). Unlike the LH sampling site, the grasses zone along the slope at PK may hinder the deposition process due to their fine and dense root systems, which likely shift to topsoil layer to assimilate water during droughts (Weides et al., 2024), the direct negative effect of drought on soil multi-nutrient cycling was specifically observed at PK (**Figure 4C**).

### 4.2 Bacterial community composition matters more than diversity in influencing the soil multi-nutrient cycling under drought in ecological buffer zones

Contrasting with the increase in soil multi-nutrient cycling, bacterial α-diversity was significantly decreased in vegetated soils under drought conditions (**Figure 2A**). This finding aligns with previous extensive studies ranging from global to regional scales, which suggest that microbial diversity decreases with increasing aridity (Maestre et al., 2015; Jiao et al., 2022b; Zhang et al., 2024b). Droughted environments, often characterized by low soil SWC and sparse above-ground communities, lead to increased niche overlaps (e.g., microbial competition), resulting in less diverse microbial communities (Zhang et al., 2022b). However, bacterial α-diversity in bare soil at both sites remained unchanged by drought but decreased significantly in these vegetated soils with relatively higher SWC and nutrient contents. Indeed, the composition of rhizodeposits actually varies among plant species, which in turn selectively recruit microbial populations with specific structures and functions that foster favorable conditions for plant growth, development, and stress tolerance (Berg and Smalla, 2009; Preece and Peñuelas, 2016). This was supported by the clear differentiation of bacterial communities and enrichment of specific bacterial taxa at vegetated soils (**Figures 2D–H**), as well as the strong effects of vegetation on shaping and selecting bacterial communities (**Figures 4A, B, D**). Nevertheless, Preece et al. (2019) documented that the water-stress effect on microbial diversity was not affected by the presence of olm oak (*Quercus ilex* L.). This discrepancy likely arises from differences in plant species characteristics, the severity and duration of drought, and other contextual environmental factors, highlighting the intricate nature of plant-microbe interactions and their variable responses to drought conditions.

Higher microbial diversity promotes multifunctionality in natural terrestrial ecosystems and moderates the resistance of multifunctionality to climate change. However. this facilitative relationship is context dependent across complex ecosystems (Hu et al., 2021; Jiao et al., 2021). For example, the linkages between biodiversity and multifunctionality have been reported to weaken or strengthen with increasing aridity in different ecosystems (Hu et al., 2021; Zhang et al., 2024a). Our SEM analysis showed the significant influence of community composition on soil multi-nutrient cycling at both sites, underscoring the crucial role of bacterial community composition in soil nutrient cycling. In a wetland ecosystem exposed to hydrological changes, Sun et al. (2024) found that bacterial β-diversity, rather than α-diversity, strongly correlated with soil multi-nutrient cycling. Studies have also shown that soil multifunctionality resistance to climate change and fertilizer input is regulated by soil bacterial communities in natural and agricultural ecosystems (Jiao et al., 2021; Luo et al., 2023). Together, our results support the view that microbial β-diversity matters more than α-diversity in the provisioning of ecosystem functions (Mori et al., 2018), which advances our knowledge of the biodiversity-function relationship in the ecological buffer zones under drought stress along YRB.

On the other hand, soil nutrient contents and other environmental variables can, in turn, affect microbial community structure. Our results showed that plant presence significantly influenced bacterial communities more than direct changes in SWC during drought (**Figures 4A, B**), which partly supported our hypothesis that drought effects would be mediated by the presence of plants. Interestingly, soil TN content, a key predictor of soil multi-nutrient cycling capacity (**Figures 3C, D**), did not directly affect bacterial communities in a significant way. This possibly because the high growth rate and/or rapid resilient ability of functional microbes associated with N-cycling under the drought in the ecological buffer zones (Allison and Martiny, 2008). Additionally, soil pH, which typically relates to substrate and nutrient availability and influences microbial structure and soil C- and N-cycling processes (Luo et al., 2023), showed significant effects on bacterial communities at both sites. However, it had a lesser impact on soil multi-nutrient cycling, possibly due to the dominant role of bacterial communities and other factors (e.g., TN) that masked pH effects. While our SEM analyses explained the majority of MNCI variation (**Figures 4C, D**), it is important to note that the relationships between soil properties and microorganisms are reciprocal (Philippot et al., 2024). Specifically, microorganisms selected by drought conditions can drive environmental changes, which in turn affect microbial community composition, activity, and evolutionary trajectories (Philippot et al., 2024).

### 4.3 Indicators and drivers of drought-affected soil multi-nutrient cycling by key bacterial taxa

In this study, the LEfSe was conducted to identify the biomarkers from the high (phylum) to low (genus) taxonomic resolution that exhibited significant differences among zones, with further assessment of their roles as an indicator of soil multi-nutrient cycling. The results showed that several important biomarkers, such as c_Bacilli, f_ Nitrosomonadaceae and g_MND1 were sensitive to increasing soil multi-nutrient cycling at PK (**Figure 3A**). Bacilli, known for their prevalence in arable soils under drought stress (Santos-Medellín et al., 2017), suggest their capability for drought resistance. While multiple species within Bacilli (e.g., *Bacilli* spp.) can confer drought tolerance to the host, their lower abundance in vegetated soils here implies they are not recruited by plants. The Nitrosomonadaceae, including g_MND1, are ammonia oxidizing bacteria that convert reduced N from ammonium/ammonia forms to nitrite (Wu et al., 2021). Their enrichment in bare soils may imply the stimulation of soil N-cycling processes by drought. In contrast, the top biomarkers identified at LH were all enriched in vegetated soils (especially at MTS), which promoted soil multi-nutrient cycling (**Figure 2H**). Notably, most of these biomarkers belong to the phylum Chloroflexi, encompassing c_Anaerolineae, o_Anaerolineales and f_Anaerolineaceae. Previous studies have shown that Chloroflexi is the aerobic/anaerobic thermophilic bacteria that grows well under drought environments (Yamada et al., 2005; Ullah et al., 2019). This finding may suggest that within the ecological buffer zones along YRB, plants can recruit the Anaerolineae to withstand drought, evident from their consistent enrichment in vegetated soils at both sites, thereby aiding to sustain various physiological functions and the growth of plants (Santos-Medellín et al., 2017; Ullah et al., 2019).

On the other hand, the bacterial communities within the key modules of networks, rather than their diversity, appeared to largely support higher soil multi-nutrient cycling (47% - 63%) (**Figure 5**, **Supplementary Figures S3A, B**), validating the idea that “community composition mattered more” as outlined earlier. Further, the random forest and regression modeling analyses were used to identify the bacterial taxa (at OTU-level) as key drivers for soil multi-nutrient cycling (**Figure 6**). These identified taxa were intricately involved deeply in soil nutrient cycling processes, especially in N-cycling including OTU8713 (f_ B1-7BS; Yu et al., 2022), OTU9465 (g_Nocardioides; Ikunaga et al., 2011), OTU:8770 (o_ SBR1031; Li et al., 2020) and OTU9168 (f_ A0839; Rodriguez-Sanchez et al., 2019). This finding suggests that drought-induced changes in functional groups (i.e., N-cycling) are important predictors of ecosystem functioning, and more accurately reflect the biological mechanisms through which microbial communities influence ecosystem functions (Osburn et al., 2023). The significant role of TN in influencing soil multi-nutrient cycling further supports these assumptions (**Figures 3C, D**).

### 4.4 Implications and limitations

Contrasting with the extensive body of studies utilizing artificially manipulated drought conditions (e.g., rain removal and warming), this study revealed changes in microbial community structure and its associated soil multi-nutrient cycling in the ecological buffer zones of YRB in the context of the 2022 extreme-drought event along the YRB. Our findings underscore the complex interactions among soil properties, plant, bacterial communities, and their combined influence on soil multi-nutrient cycling upon the drought event, highlighting the distinct mechanisms at play in response to extreme environmental stress in ecological buffer zones. For example, the drought effects on bacterial community diversity and soil multi-nutrient cycling are modified by the presence of plants, varying between bare and vegetated soils, possibly as a result of extreme drought leading to the release of more rhizodeposits by vegetation, and in turn selectively recruiting bacterial communities. This contrasted slightly with early findings from a culture experiment exposed to water stress, suggesting that the presence of the plant on microbial communities would be very minor, especially when compared with water stress or soil history (Preece et al., 2019).

However, it should be noted that due to the general dominance of a single species in the ecological zones (**Supplementary Figure S1**), this study cannot test whether the presence of diverse plants mitigates the selection effects of the single species on microbial communities against the extreme drought, with high-diversity communities being more resistant to climate change (Steudel et al., 2012; Jiao et al., 2022b). Looking forward, the establishment and development of national nature reserves along YRB could provide an excellent opportunity for future study on the interaction of plant diversity and drought, especially given the increasing frequency of extreme drought events (e.g., flash drought; Yuan et al., 2023). Additionally, future research should continue to explore the interactive effects of plant-microbial interactions and soil properties on nutrient cycling dynamics across varying spatial and temporal scales to enhance our understanding and management of ecosystem resilience in the face of climate change.

## 5 Conclusion

This study revealed that in the ecological buffer zones along the Yangtze River, the extreme drought induced a significant decrease in bacterial α-diversity but an increase in the soil nutrient cycling index (MNCI) in vegetated soils, with non-significant changes in bare soils. This suggested that the effects of drought could be modified by the presence of plants. Soil bacterial community composition (β-diversity) exhibited a more important role than α-diversity in influencing MNCI. The key ecological clusters (Module 1) within co-occurrence networks at both sites were significantly positively correlated with MNCI, even though the network structure (involving nodes, edges and average degree) was not substantially changed by the drought. Furthermore, the strongest predictors (at OTU-level) for MNCI within Module 1 could be deeply involved in soil N-cycling. Collectively, this study advances the knowledge of the association between soil microbial communities and multifunctionality as affected by the natural extreme drought event in the ecological buffer zones along the Yangtze River, and thus aiding to forecast the ecological consequences arising from increasingly frequent global extreme drought event.

## Data Availability

The datasets presented in this study can be found in online repositories. The names of the repository/repositories and accession number(s) can be found below: https://www.ncbi.nlm.nih.gov/, PRJNA1139050.
