# Supplementary material for: Key microbial taxa play essential roles in maintaining soil muti-nutrient cycling following an extreme drought event in ecological buffer zones along the Yangtze River
Source: Front Plant Sci. 2024 Sep 4;15:1460462. doi: 10.3389/fpls.2024.1460462 (PMC11408313; doi:10.3389/fpls.2024.1460462)
Supplement: Supplementary file 1 [file DataSheet1.pdf]

Supporting Information For:

Key microbial taxa play essential roles in maintaining soil muti-nutrient cycling following an extreme drought event in ecological buffer zones along the Yangtze River

Jie Fang [a#], Zihao Liu [a#], Yongcui Deng [b], Bin Song [a,c,*], Jonathan M. Adams [a,*]

[a] School of Geography and Ocean Sciences, Nanjing University, Nanjing 210008, China

[b] School of Geography, Nanjing Normal University, Nanjing 210008, China

[c] Department of Forest Sciences, University of Helsinki, PO Box 27, Latokartanonkaari 7, FI-00014 Helsinki, Finland

#These authors contributed equally to this work

(🖂) Corresponding authors:

Dr. Bin Song

E-mail: bsong@nju.edu.cn

Dr. Jonathan M. Adams

E-mail: jonadams@nju.edu.cn

Table S1 Soil properties and enzyme activities along zones at sampling site of Pukou (PK) district

	CK	BS	YRS	GSS	ORS
SWC (%)	35.9±2.4ab	27.3±1.2c	31.8±1.7b	32.4±1.5b	38.2±2.7a
Conduc (ms/cm)	169±6b	180±27b	315±108b	491±73b	915±407a
Sal (g/L)	0.08±0.00b	0.09±0.1b	0.15±0.06b	0.23±0.03b	0.45±0.20a
pH	8.11±0.06a	8.11±0.12a	7.88±0.14b	7.74±0.06bc	7.66±0.14c
TC (g/kg)	15.3±0.9c	15.4±1.1c	17.0±1.2c	20.5±3.5b	27.1±0.2a
TN (g/kg)	0.9±0.1d	1.0±0.0d	1.4±0.2c	1.8±0.4b	2.4±0.1a
TP (g/kg)	0.8±0.0c	0.9±0.0bc	0.9±0.0bc	0.9±0.1ab	1.0±0.1a
AN (mg/kg)	78.2±8.4c	67.2±7.1c	108.3±21.3c	167.4±46.7b	231.5±29.5a
AP (mg/kg)	26.5±0.4b	25.0±3.3b	29.8±3.2ab	38.2±9.4a	27.9±2.0b
BG (nmol $g^{-1}h^{-1}$)	11.6±3.7b	9.6±1.4b	28.2±13.1b	30.6±13.9b	66.3±15.4a
BX (nmol $g^{-1}h^{-1}$)	2.7±1.2bc	0.8±0.9c	4.8±2.3ab	3.8±1.3bc	7.9±2.9a
LAP (nmol g^{-1} h^{-1})	15.1±2.5a	3.8±1.0b	13.2±3.3a	17.1±5.2a	17.5±0.5a
NAG (nmol g^{-1}h-1)	6.2±1.9b	4.9±1.4b	13.1±7.0b	14.0±5.1b	32.0±7.5a
ALP (nmol g-1h-1)	30.1±4.9bc	21.7±6.9c	60.1±14.0bc	75.6±21.8ab	113.0±50.6a

Values are the means with standard deviations (n = 3). CK: moist soil near the river; BS: bare soil; YRS: soil with young reeds; GSS: soil with grasses; ORS: soil with old reeds; SWC: soil water content; Conduc: conductivity; Sal: salinity; TC: total carbon; TN: total nitrogen; TP: total phosphorus; AN: available nitrogen; AP: available phosphorus; BG: β-glucosidase; BX: β-Xylosidase; LAP: leucine aminopeptidase; NAG: N-acetyl-β-glucosaminidase; ALP: alkaline phosphatase.

Table S2 Soil properties and enzyme activities along zones at sampling site of Luhe (LH) district

	CK	BS	YRS	ORS	MTS
SWC (%)	33.0±0.5a	28.2±3.0ab	29.7±1.2ab	29.8±9.3ab	21.6±2.4b
Conduc (ms/cm)	177±30b	233±24b	340±98ab	513±290a	338±13ab
Sal (g/L)	0.09±0.01b	0.11±0.01b	0.17±0.05b	0.25±0.14b	0.17±0.01a
pH	8.12±0.09a	8.04±0.18ab	7.89±0.16ab	7.79±0.23b	7.81±0.08b
TC (g/kg)	13.5±0.4b	14.8±0.7ab	16.4±0.4a	17.3±2.6a	16.2±1.0a
TN (g/kg)	0.6±0.3b	0.9±0.0ab	1.1±0.1a	1.2±0.4a	1.1±0.1a
TP (g/kg)	0.77±0.02cd	0.76±0.00d	0.81±0.02b	0.85±0.01a	0.80±0.00bc
AN (mg/kg)	61.4±25.6b	97.7±38.8ab	104.7±7.2ab	138.2±37.3a	109.0±31.9ab
AP (mg/kg)	18.3±6.0b	23.4±1.1ab	26.2±1.5a	24.1±5.3ab	17.4±1.0b
BG (nmol $g^{-1}h^{-1}$)	10.8±4.4b	11.0±3.0b	17.6±7.2b	31.4±19.1ab	45.1±22.8a
BX (nmol $g^{-1}h^{-1}$)	1.2±0.8b	1.1±0.4b	3.0±1.9b	6.9±3.1a	2.8±0.3b
LAP (nmol g^{-1} h^{-1})	9.3±7.0c	3.9±2.8c	11.1±6.5bc	29.3±12.0a	23.8±6.5ab
NAG (nmol $g^{-1}h^{-1}$)	4.7±1.4b	5.6±1.3ab	11.0±7.6ab	19.7±15.2ab	24.8±14.2a
ALP (nmol $g^{-1}h^{-1}$)	17.7±6.1b	23.1±13.2b	51.7±29.7ab	84.8±47.4a	77.1±14.8a

Values are the means with standard deviations (n = 3). CK: wetting soil near the river; BS: bare soil; YRS: soil with young reeds; ORS: soil with old reeds; MTS: soils with mixed trees; SWC: soil water content; Conduc: conductivity; Sal: salinity; TC: total carbon; TN: total nitrogen; TP: total phosphorus; AN: available nitrogen; AP: available phosphorus; BG: β-glucosidase; BX:β-Xylosidase; LAP: leucine aminopeptidase; NAG: N-acetyl-β-glucosaminidase; ALP: alkaline phosphatase.

Table S3 Pairwise comparison between zones by PERMANOVA

**PK:**	R^2	*p*
CK vs BS	0.327	0.100
CK vs Vegetated soils	0.386	0.004
BS vs Vegetated soils	0.357	0.008
**LH:**		
CK vs BS	0.213	0.400
CK vs Vegetated soils	0.227	0.034
BS vs Vegetated soils	0.226	0.026

In PK sampling site, the vegetated soils including soil with young reeds (YRS); soil with grasses (GSS) and soil with old reeds (ORS). In LH sampling site, the vegetated soils including soil with young reeds (YRS), soil with old reeds (ORS) and soil with mixed trees (MTS).

Table S4 Network topological features across zones at sampling sites of Pukou (PK) and Luhe (LH) district

**PK:**	Nodes	Edges	Average degree
CK	384	14753	76.77
BS	371	13488	72.48
YRS	385	14752	76.57
GSS	385	14621	75.88
ORS	378	13808	73.05
**LH:**			
CK	392	19806	101.05
BS	395	20066	101.68
YRS	396	20088	101.46
ORS	378	17882	94.115
MTS	381	18200	95.31

CK: wetting soil near the river; BS: bare soil; YRS: soil with young reeds; GSS: soil with grasses ORS: soil with old reeds; MTS: soils with mixed trees;

Fig. S1 Example of a sampling site along Yangtze River in Luhe district. The right of the dotted line is the wetting soil in the waterward zone, which was virtually unaffected by drought and was collected as the ambient soil (i.e., the control), given the fact that topsoil tended to be homogenized by the reciprocating tidal flows in limited successional zones within the sample site during the regular period.

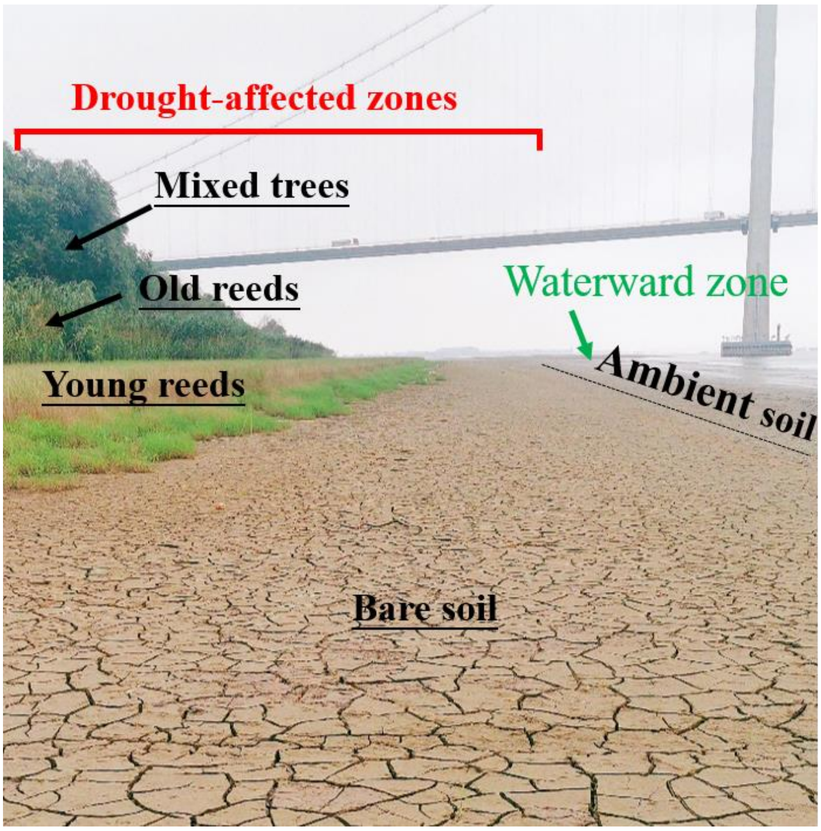

Fig. S2 ZP-plot showing distributions of nodes based on their network roles at PK (A) and LH (B). Zi: within-module connectivity; Pi: among-module connectivity. The topological role of each node in the networks was determined by Zi and Pi. Nodes were defined as module hubs (Zi > 2.5, Pi < 0.62; (nodes highly connected within a module), network hubs (Zi > 2.5, Pi > 0.62; nodes highly connected both within and between modules), peripherals (Zi < 2.5, Pi <0.62; few connected nodes between modules) and connectors (Zi < 2.5; Pi >0.62; highly connected nodes between modules) (Olesen et al., 2007; Deng et al., 2012), and keystone taxa were described as module hubs or connectors in the networks (Zhou et al., 2011). Each node represents a bacterial OTU.

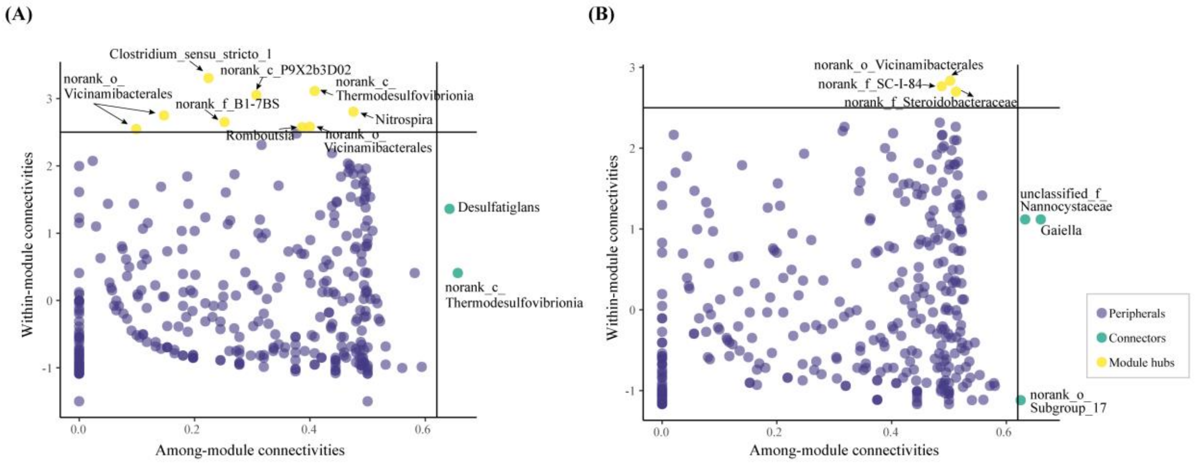

Fig. 3 Relationships between bacterial diversity within Module 1 and soil multi-nutrient cycling index at PK and LH (A&B). ρ indicates Spearman's correlation coefficients, with ρ > 0.05 as nonsignificant relationships. Ternary plot of OTUs distribution across ecological zones at PK and LH (C&D), respectively. Each circle indicates one OTU, and the position, size and color of each circle indicate its significant enrichment in specific zones, relative abundance and bacterial phylum, respectively. Grey circles indicate no significant differences in abundance, and the number in parentheses at each vertex indicates the number of enriched OTUs.

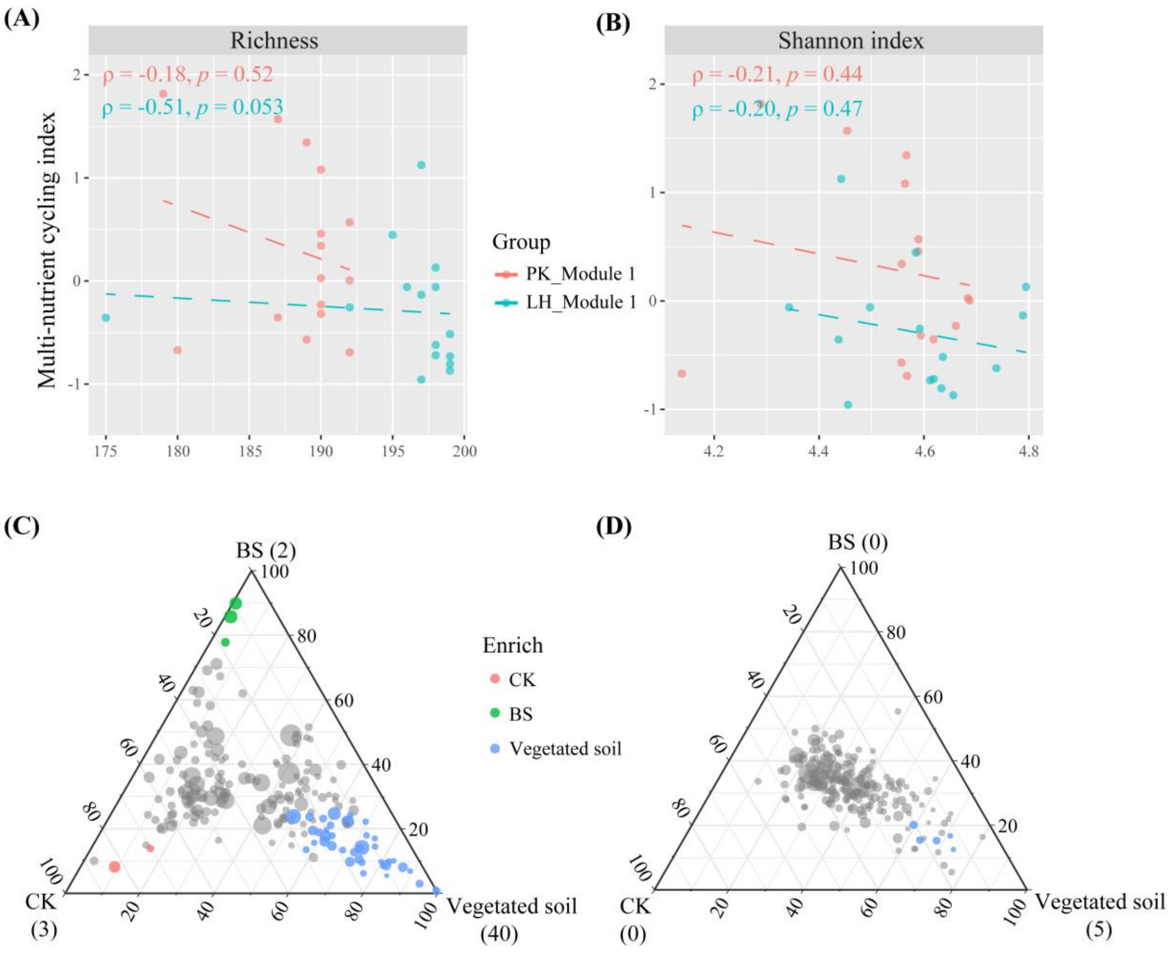

Fig. S4 Linear discriminant analysis (LDA) effect size analysis of relative abundances of predicted functions (by Tax4Fun2) of bacterial communities within Module 1 of network at LH. All differentiated functions were shown (LDA > 2). a, b, c indicate the predicted functions at level 1, level 2 and level 3, respectively. CK: wetting soil near the river; BS: bare soil; YRS: soil with young reeds; MTS: soils with mixed trees.

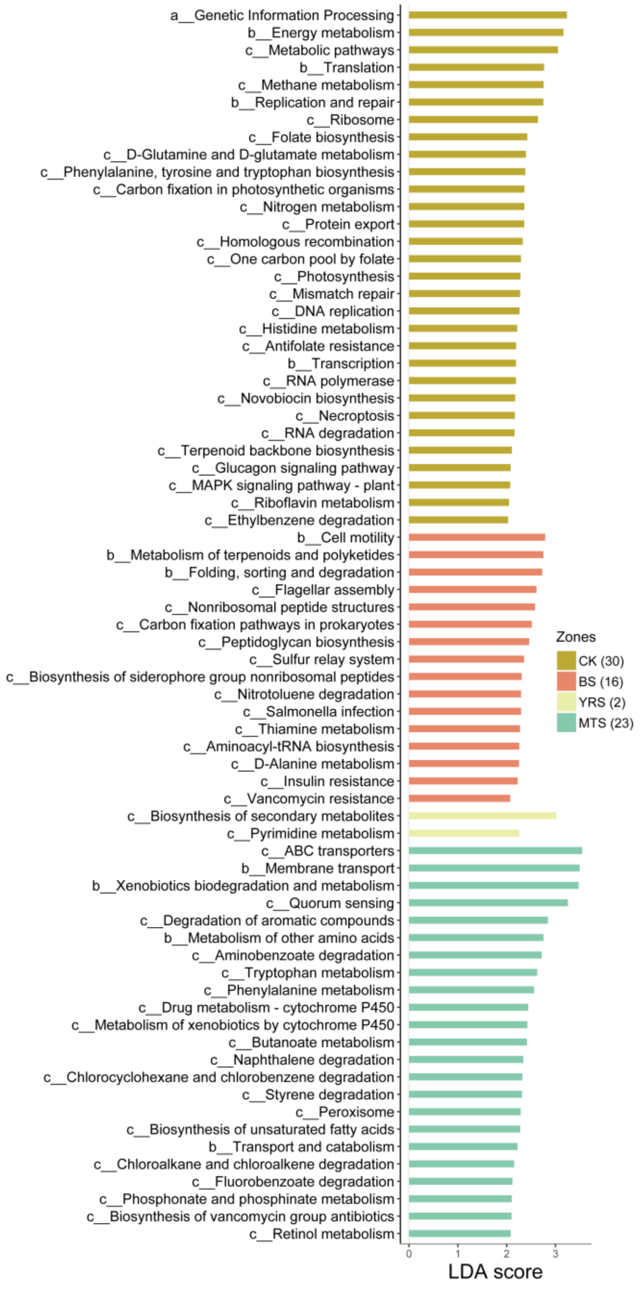

References:

Deng, Y., Jiang, Y.H., Yang, Y., He, Z., Luo, F., Zhou, J., 2012. Molecular ecological network analyses. BMC bioinformatics 13, 1-20.

Olesen, J.M., Bascompte, J., Dupont, Y.L., Jordano, P., 2007. The modularity of pollination networks. Proceedings of the National Academy of Sciences 104, 19891-19896.

Zhou, J., Deng, Y., Luo, F., He, Z., Yang, Y., 2011. Phylogenetic molecular ecological network of soil microbial communities in response to elevated CO_2. mBio 2, e00122-11.
